# IPSC reprogramming of two patients with spondyloepimetaphyseal dysplasia (SEMD, biglycan type)

**DOI:** 10.1016/j.scr.2023.103024

**Published:** 2023-03

**Authors:** Pauline De Kinderen, Silke Peeters, Laura Rabaut, Geert Mortier, Peter Ponsaerts, Bart Loeys, Aline Verstraeten, Josephina A.N. Meester

**Affiliations:** a Center of Medical Genetics, University of Antwerp and Antwerp University Hospital, Antwerp Belgium; b Center of Human Genetics, KU Leuven and Leuven University Hospital, Leuven, Belgium; c Laboratory of Experimental Hematology, Vaccine and Infectious Disease Institute (Vaxinfectio), University of Antwerp, Antwerp, Belgium; d Department of Clinical Genetics, Radboud University Medical Center, Nijmegen, the Netherlands

## Abstract

Hemizygous missense variants in the X-linked *BGN* gene, encoding the extracellular matrix protein biglycan, cause spondyloepimetaphyseal dysplasia (SEMD, biglycan type), which is clinically characterized by short stature, brachydactyly and osteoarthritis. Little is known about the pathomechanisms underlying SEMD, biglycan type. IPSC-derived chondrocyte disease models have been shown to exhibit several key aspects of known disease mechanisms of skeletal dysplasias and are therefore considered highly suitable human disease models to study SEMD, biglycan type. Prior to creating iPSC-chondrocytes, dermal fibroblasts of two male patients with SEMD, biglycan type, carrying the p.Gly259Val variant were successfully reprogrammed into iPSCs using the CytoTune^TM^-iPS 2.0 Sendai Kit (Invitrogen).

## 1 Introduction

### 1.1 Resource Table:

**Table:** 

Unique stem cell lines identifier	CMGANTi003-A CMGANTi004-A
Alternative name(s) of stem cell lines	SEMD1 (CMGANTi003-A)SEMD2 (CMGANTi004-A)
Institution	University of Antwerp and Antwerp University Hospital
Contact information of distributor	Josephina Meester - Josephina.Meester@uantwerpen.be
Type of cell lines	iPSC
Origin	human
Additional origin info required	CMGANTi003-A: 52 yrs, male, Italian CMGANTi004-A: 50 yrs, male, Italian
Cell Source	dermal fibroblasts
Clonality	clonal
Method of reprogramming	Sendai virus
Genetic Modification	yes
Type of Genetic Modification	hereditary
Evidence of the reprogramming transgene loss (including genomic copy if applicable)	Absence of the Sendai virus backbone was verified with PCR and agarose gel electrophoresis.
Associated disease	spondyloepimetaphyseal dysplasia (SEMD), biglycan type
Gene/locus	*BGN* c.776G > T (NM_001711)
Date archived/stock date	December 2020
Cell line repository/bank	Hpscreg https://hpscreg.eu/cell-line/CMGANTi003-A https://hpscreg.eu/cell-line/CMGANTi004-A
Ethical approval	Ethical committee Antwerp University Hospital, approval number: 11/8/79 2018.09.17

## 2 Resource utility

Because a cartilage biopsy is a highly invasive procedure for the patient and the regenerative capacity of cartilage tissue is limited, iPSC-derived chondrocytes provide a valuable alternative to model chondrodysplasias, including SEMD, biglycan type, and to investigate the underlying pathomechanisms.

## 3 Resource details

**Fig. 1:**
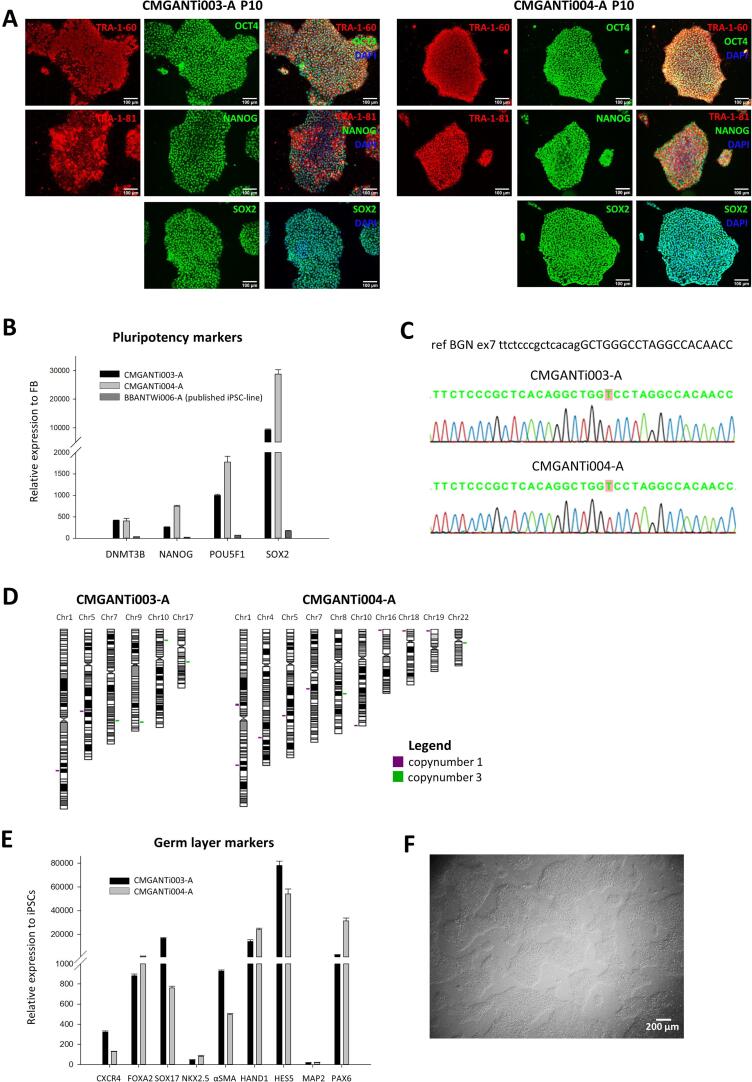
Characterization of iPSC-line CMGANTi003-A and CMGANTi004-A.

**Supplementary figure 1:**
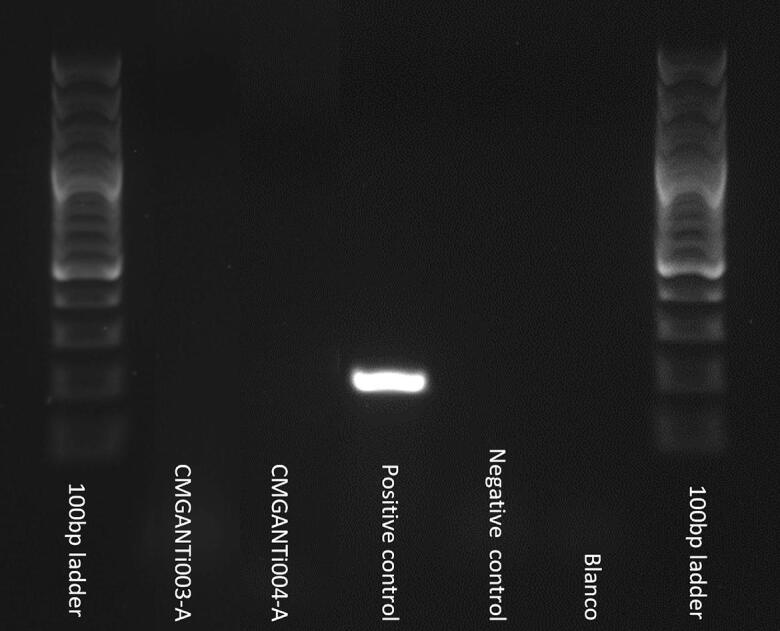
Loss of Sendai viral vector.

**Supplementary figure 2:**
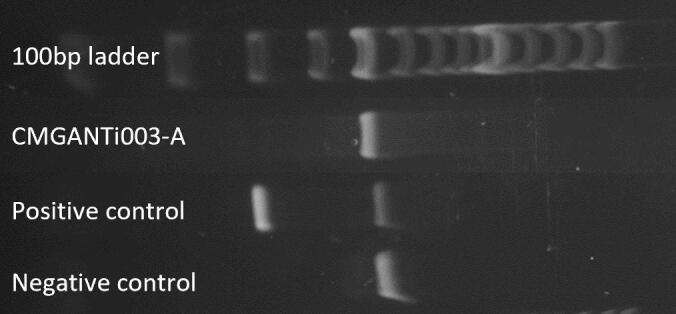
Absence of mycoplasma CMGANTi003-A.

**Supplementary figure 3:**
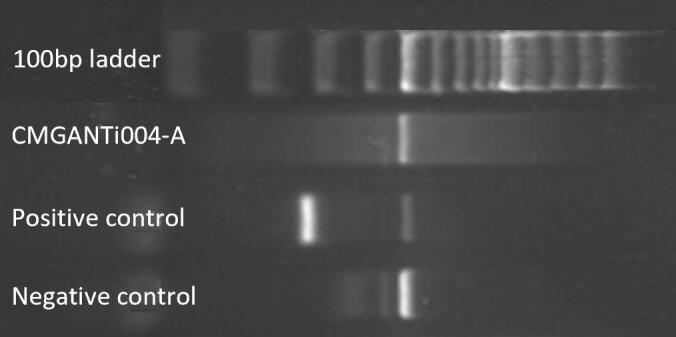
Absence of mycoplasma CMGANTi004-A.

Specific hemizygous missense variants in the X-linked *BGN* gene, encoding the extracellular matrix protein biglycan, cause spondyloepimetaphyseal dysplasia (SEMD, biglycan type) (Cho et al., 2016), while loss-of-function pathogenic variants in this gene have been linked to an aortopathy syndrome called Meester-Loeys Syndrome (Meester et al., 2017). Clinical features of SEMD, biglycan type include short stature, brachydactyly and osteoarthritis. Some symptoms can be treated with surgery (e.g. short limbs). However, this is not without complications (e.g. risk of nerve injury), comes with high medical costs and is associated with a painful revalidation. There is thus clearly a need for curative treatments addressing the underlying pathophysiology. Little is known about the pathomechanisms underlying SEMD, biglycan type. To improve the current understanding, it is key to develop a representative human disease model. Induced pluripotent stem cell (iPSC)-derived chondrocyte disease models have been shown to exhibit several key aspects of the known disease mechanisms of skeletal dysplasias and are therefore considered highly suitable. Prior to creating iPSC-chondrocyte disease models, iPSCs need to be generated. In this article, we introduce two SEMD, biglycan iPSC-lines of two Italian brothers both carrying a missense variant in the *BGN* gene (p.Gly259Val). Dermal fibroblasts of the two male SEMD, biglycan type patients were reprogrammed into iPSCs using the CytoTyne^TM^-iPS 2.0 Sendai Kit (Invitrogen). This kit contains three Sendai viral reprogramming vectors delivering and expressing the key genetic factors OCT3/4, SOX2, KLF4 and c-MYC necessary for iPSC generation from somatic cells. Pluripotency of the resulting iPSCs was confirmed using immunocytochemistry (ICC) for the pluripotency markers OCT4, SOX2, NANOG, TRA-1–60 and TRA-1–81 (Fig. 1, A) and real-time quantitative polymerase chain reaction (RT-qPCR) for expression levels of NANOG, POU5F1, DNTM3B and SOX2 (Fig. 1, B). The iPSCs were able to differentiate into the three germ layers, *i.e.* ectoderm, mesoderm and endoderm, which was proven using RT-qPCR for appropriate markers of the respective germ layers (Fig. 1, E). Presence of the pathogenic variant in both iPSC lines was confirmed using Sanger sequencing (Fig. 1, C). Copy number variation (CNV) analysis using single nucleotide polymorphism (SNP) arrays verified genomic identity between the created iPSC clones and the original fibroblast cell line (Table 3). Genomic stability of the iPSC clones and the original fibroblasts was also investigated by a CNV analysis. No indels were observed in genes described in the ‘Nosology and classification of genetic skeletal disorders: 2019 revision’ of *Mortier et al.* (Fig. 1, D (duplications in green, deletions in purple) and Supplementary file 1) (Mortier et al., 2019). Therefore, it can be concluded that no clinically relevant CNVs were introduced during the reprogramming process. A more detailed overview of these CNVs and the involved genes can be found in Supplementary file 1. Note that this SNP array is not able to detect balanced rearrangements and low-level mosaicism. Furthermore, the iPSC clones were free of the Sendai viral backbone (Supplementary Figure 1) and mycoplasma contamination (Supplementary Figure 2, Supplementary Figure 3). In conclusion, we have successfully established two patient iPSC lines as a first step in the creation of iPSC-chondrocyte models to study and therapeutically target the disease mechanisms underlying SEMD, biglycan type.

## 4 Materials and methods

### 4.1 Fibroblast origin & culture and iPSC reprogramming

Dermal fibroblasts of the two SEMD, biglycan type patients were acquired from the Galliera Genetic Bank (Baldo et al., 2016). They were cultured in RPMI medium (Life Technologies) supplemented with 15 % FBS (Life Technologies), 1 % sodium pyruvate (Life Technologies), 100 U/mL Pen/Strep (Life Technologies) and 0.1 % primocin (InvivoGen Europe). The fibroblasts were reprogrammed into iPSCs using the CytoTune^TM^-iPS 2.0 Sendai Kit (Life Technologies) according to the manufacturer’s protocol. In short, fibroblasts were transduced by three reprogramming vectors, which express the key genetic factors necessary for iPSC generation (*i.e.* OCT3/4, SOX2, KLF4 and c-MYC). Seven days after transduction, the cells were transferred to Matrigel coating (Corning) and 24 h later the medium was changed into iPSC medium. IPSC colonies were manually picked and further expanded by passaging the cells as small clumps every 4–5 days (1:5 ratio) using 0.02 % EDTA in Essential 8^TM^ Flex medium (Life Technologies) supplemented with RevitaCell (Life Technologies) on Matrigel-coated dishes at 37 °C, 5 % CO_2_, 5 % O_2_.

### 4.2 Immunocytochemistry

After reprogramming, the iPSCs (passage 10) were cultured on coverslips and fixed with 100 % methanol (20′, −20 °C). Then, they were permeabilized using 0.1 % Triton X-100 solution (Sigma-Aldrich) (15′, room temperature (RT)). Non-specific binding was blocked using 5 % goat serum (Jackson ImmunoResearch) (30′, RT) and the primary antibodies were added and incubated overnight (4 °C). Hereafter, the cells were washed using 0.1 % Triton X-100 and secondary antibodies were incubated for one hour (RT). DAPI (Life Technologies) was used to visualize the cell nuclei. Coverslips were mounted on glass slides and pictures were taken using a 20x objective from Olympus BX51 fluorescence microscope.

### 4.3 Quantitative pluripotency marker analysis

RNA was extracted from both fibroblast and iPSC cell pellets (passage 10) using the Quick-RNA^TM^ Miniprep Kit (ZYMO Research). Subsequently, cDNA was synthesized using the SuperScript^TM^ III First-Strand Synthesis System (Life Technologies). Expression of the selected pluripotency markers (Table 1) was confirmed using RT-qPCR TaqMan® probes (Life Technologies) (Table 2) using a BioRad CFX384 Real-Time system (50 °C 2′, 95 °C 10′, 40x (95 °C 15′', 60 °C 1′)).

**Table 1:** Characterization and validation.

**Classification**	**Test**	**Result**	**Data**
**Morphology**	Photography Bright field	Normal	Fig. 1 panel F
**Phenotype**	Qualitative analysis (Immunocytochemistry)	Staining/expression of pluripotency markers: Oct3/4, Nanog, Sox2, Tra1-60, Tra1-80.	Fig. 1 panel A
	Quantitative analysis (RT-qPCR)	Expression of DNMT3B, NANOG, POU5F1 and SOX2	Fig. 1 panel B
**Genotype**	HumanCytoSNP-12 array	Resolution 72 kb, no major copy number variations	Fig. 1 panel D
**Identity**	HumanCytoSNP-12 array OR	> 99.9 % identical SNPs	Table 3
	STR analysis	N/A	N/A
**Mutation analysis (IF APPLICABLE)**	Sequencing	Hemizygous *BGN* c.776G > T	Fig. 1 panel C
	Southern Blot OR WGS	N/A	N/A
**Microbiology and virology**	Mycoplasma	Negative	Supplementary Fig. 2, Supplementary Fig. 3
**Differentiation potential**	Trilineage differentiation	Expression of appropriate markers of the respective germ layers, *i.e.* ectoderm, mesoderm and endoderm.	Fig. 1 panel E
**List of recommended germ layer markers**	**Expression of these markers has to be demonstrated at mRNA (RT PCR) or protein (IF) levels, at least 2 markers need to be shown per germ layer**	Endoderm: *CXCR4, FOXA2*, *SOX17* Mesoderm: *NKX2.5, αSMA (ACTA2*), *HAND1* Ectoderm: *HES5*, *MAP2*, *PAX6*	Fig. 1 panel E
**Donor screening (OPTIONAL)**	HIV 1 + 2 Hepatitis B, Hepatitis C	N/A	N/A
**Genotype additional info (OPTIONAL)**	Blood group genotyping	N/A	N/A
	HLA tissue typing	N/A	N/A

**Table 2:** Reagents details.

	**Antibodies used for immunocytochemistry/flow-cytometry**			
	**Antibody**	**Dilution**	**Company Cat #**	**RRID**
Pluripotency Markers	Mouse anti-TRA1-60	1:200	Cell Signaling Technology Cat#4746S	AB_2119059
	Rabbit anti-OCT4	1:100	Thermo Fisher Scientific Cat#PA596860	AB_2808662
	Rabbit anti-SOX2	1:500	Merck Millipore Cat#AB5603	AB_2286686
	Mouse anti-TRA1-81	1:200	Cell Signaling Technology Cat#4745S	AB_2119060
	Rabbit anti-NANOG	1:500	ThermoFisher Scientific Cat#PA1-097	AB_2539867
Secondary antibodies	AF555 Goat anti-Mouse, IgM	1:500	Thermo Fisher Scientific Cat#A21426	AB_2535847
	AF488 Goat anti-Rabbit, IgG	1:500	Thermo Fisher scientific Cat#A11034	AB_2576217

	**Primers**			
	**Target**	**Size of band**	**Forward/Reverse primer (5′-3′)**	

Pluripotency Markers (RT-qPCR)	*DNMT3B*	55 bp	Hs00171876_m1	
	*NANOG*	99 bp	Hs04260366_g1	
	*POU5F1*	77 bp	Hs04260367_gH	
	*SOX2*	91 bp	Hs01053049_s1	
House-Keeping Genes (RT-qPCR)	*GAPDH*	93 bp	Hs02758991_g1	
	*ACTB*	63 bp	Hs01060665_g1	
Differentiation markers (RT-qPCR)	*CXCR4*	153 bp	Hs00607978_s1	
	*FOXA2*	66 bp	Hs00232764_m1	
	*SOX17*	149 bp	Hs00751752_s1	
	*NKX2.5*	64 bp	Hs00231763_m1	
	*αSMA (ACTA2)*	105 bp	Hs00426835_g1	
	*HAND1*	54 bp	Hs00231848_m1	
	*HES5*	62 bp	Hs01387463_g1	
	*MAP2*	98 bp	Hs00258900_m1	
	*PAX6*	76 bp	Hs00240871_m1	
Targeted mutation sequencing	*BGN* c.776G > T	319 bp	GTTTTCCCAGTCACGACAAGGGTGATGCCAGAGTCC/ CAGGAAACAGCTATGACGACTGAGGGACTGCCCG	
Sendai virus Plasmids (PCR)	SeV	181 bp	GGATCACTAGGTGATATCGAGC/ACCAGACAAGAGTTTAAGAGATATGTATC	
	KOS	528 bp	ATGCACCGCTACGACGTGAGCGC/ ACCTTGACAATCCTGATGTGGyc	
	Klf4	410 bp	TTCCTGCATGCCAGAGGAGCCC/AATGTATCGAAGGTGCTCAA	
	c-Myc	532 bp	TAACTGACTAGCAGGCTTGTCG/ TCCACATACAGTCCTGGATGATGATG

**Table 3:** Cell line identity testing.

**iPSC line**	**total count**	**correct count**	**errors**	**% identical**
CMGANTi003-A P10	288,134	288,131	3	>99.9 %
CMGANTi004-A P10	287,779	287,769	10	>99.9 %

### 4.4 SNP array (CNV analysis)

Genomic DNA was isolated from the patients’ respective fibroblast and iPSC pellets (passage 10) using the Maxwell® RSC Instrument and Maxwell® RSC Cultured Cells DNA Kit (Promega) according to manufacturer’s protocol. Subsequently, a HumanCytoSNP-12 assay (Illumina) was performed according to the Infinium HD Assay Ultra Automated Protocol using an iScan System (Illumina). The obtained data was analysed in CNV-WebStore, an in-house developed online platform to analyse and interpret microarray data, to investigate the presence of CNVs between the original cell line and the created iPSC clones (Vandeweyer et al., 2011).

### 4.5 Sanger sequencing

In the genomic DNA of patient iPSCs (passage 10) and dermal fibroblasts, exon 7 of *BGN* was amplified by a Touchdown PCR (94 °C 3′, 10x (94 °C 5′, 65 °C (Δ-0.5) 15′', 72 °C 15′'), 25x (94 °C 5′, 55 °C 15′', 72 °C 15′'), 72 °C 1′)) using a Verity Thermal Cycler (Applied Biosystems). Prior to Sanger sequencing, the PCR products were purified using calf intestinal alkaline phosphatase (Merck) and Exonuclease I (BioLabs). Presence of the causal mutation was verified by Sanger sequencing reactions on an ABI 3130XL Genetic Analyzer system (Applied Biosystems) according to the standard protocol.

### 4.6 Mycoplasma test

The absence of mycoplasma in iPSC culture medium was verified using the LookOut Mycoplasma PCR Detection Kit (Sigma-Aldrich) according to the standard protocol.

### 4.7 Trilineage differentiation and analysis

To proof pluripotency, iPSCs (CMGANTi003-A passage 16 and CMGANTi004-A passage 15) were differentiated into the three embryonic germ layers (mesoderm, endoderm and ectoderm) using the StemMACS Trilineage Differentiation Kit (Miltenyi Biotec) according to manufacturer’s protocol at 37 °C, 5 % CO_2_, 20 % O_2_. On day seven, cells were collected for RNA extraction and cDNA synthesis. Expression of the selected germ layer markers (Table 1) was verified using RT-qPCR as described above (Table 2).

### 4.8 Sendai virus detection

RNA of the iPSCs (passage 10) was extracted and cDNA was synthesized as described above. Absence of the Sendai virus backbone was verified with PCR (94 °C 5′, 94 °C 15′', 34x (60 °C 30′'), 72 °C 45′', 72 °C 10′) and agarose gel electrophoresis.

## Declaration of Competing Interest

The authors declare that they have no known competing financial interests or personal relationships that could have appeared to influence the work reported in this paper.
