# Supplementary material for: IPSC reprogramming of two patients with spondyloepimetaphyseal dysplasia (SEMD, biglycan type)
Source: Stem Cell Res. 2023 Mar;67:103024. doi: 10.1016/j.scr.2023.103024 (PMC9972783; doi:10.1016/j.scr.2023.103024)
Supplement: Supplementary data 1 [file mmc1.docx]

**Supplementary file1. CNV analysis**

| **CMGANTi003-A P10** | | | | |
| --- | --- | --- | --- | --- |
| Chromosome | Copy number variation | Size | Location | Genes in region |
| 1 | deletion | 144386 | 195,763,875-195,908,262 | / |
| 5 | deletion | 35611 | 113,143,478-113,179,090 | / |
| 7 | duplication | 189792 | 125,658,470-125,848,263 | / |
| 9 | duplication | 161546 | 127,681,154-127,842,701 | GOLGA1 |
| 10 | duplication | 198551 | 14,874,692-15,073,244 | ACBD7, DCLRE1CP1, CDNF |
| 17 | duplication | 416210 | 44,163,925-44,580,136 | ARL17B, ARL17A, LRRC37A, NSFP1 |

| **CMGANTi004-A P10** | | | | |
| --- | --- | --- | --- | --- |
| Chromosome | Copy number variation | Size | Location | Genes in region |
| 1 | deletion | 88418 | 105,181,816-105,270,235 | / |
| 1 | deletion | 185207 | 190,307,072-190,492,280 | BRINP3, LINC01351 |
| 4 | deletion | 178217 | 151,275,797-151,454,015 | LRBA |
| 5 | deletion | 119734 | 120,694,906-120,814,641 | / |
| 7 | deletion | 249253 | 83,269,806-83,519,060 | SEMA3E |
| 8 | duplication | 313116 | 90,413,481-90,726,598 | / |
| 10 | deletion | 74558 | 134,529,340-134,603,899 | INPP5A |
| 16 | deletion | 61187 | 1,264,783-1,325,971 | CACNA1H |
| 18 | deletion | 97705 | 1,896,280-1,993,986 | / |
| 19 | deletion | 44736 | 1,668,161-1,712,898 | / |
| 22 | duplication | 330471 | 18,656,495-18,986,967 | DGCR5, GGT3P, LINC01662, LINC02592, LOC102725072, LOC105379550, USP18 |
